# Philip Seeman's contributions to the story of schizophrenia

**DOI:** 10.1017/S0033291721004803

**Published:** 2022-10

**Authors:** Mary V. Seeman

**Affiliations:** Department of Psychiatry, University of Toronto, 260 Heath St. West Suite $605, Toronto, ON, Canada

**Keywords:** Antipsychotics, dopamine receptors, schizophrenia, Seeman

## Abstract

Philip Seeman's isolation of the dopamine D2 receptor is an example of a small step that can lead to major change in the way that we conceptualize the etiology of schizophrenia.

It is usually impossible to attribute progress in science to any one discovery because each new step builds on incremental advances in technology and in experimental results emerging unexpectedly in various corners of the world, in disciplines not necessarily one's own. This paper tries to trace the contributions to understanding brain mechanisms leading to schizophrenia attributable to Philip Seeman, a Canadian neuropharmacologist who died on 9 January 2021. He became interested in schizophrenia in 1961 when Phil's wife, the author of this piece, began her residency in psychiatry at Manhattan State Hospital on Ward's Island in New York City. At the time, Phil was starting his PhD program at the Rockefeller Institute. He was surrounded by ‘Great Names.’ His supervisor was the already very famous George Palade, discoverer of ribosomes. Palade was later to receive the Nobel Prize in Physiology/Medicine, sharing it with Albert Claude and Christian de Duve, and awarded for their breakthrough discoveries of the components of cell structure. These discoveries were made possible by advances in the technology of the electron microscope (Oatley, 1982).

Phil Seeman had been well trained. Before receiving his medical degree from McGill University in 1960, he had completed a Master's degree, supervised by Professor Arnold Burgen, who was to become Sir Arnold in 1976, an acknowledgement of his significant work in drug-receptor interactions in the central nervous system and mechanisms of secretion. Phil's Master's thesis was on the secretion and flow of saliva. In hindsight, parallels can probably be drawn between saliva and the secretion and flow of dopamine in the brain.

At Rockefeller, students were expected to come up with their own scientific questions and pursue their own journeys of discovery. You were continually being inspired by your mentors, but there wasn't much hands-on instruction. Phil knew he wanted to use the potential of the electron microscope for his PhD; that was why he was drawn to the Palade lab. But he couldn't figure out what to use the electron microscope for. I was 6 months pregnant with our first child and hadn't yet told my residency boss that I would soon be needing a leave of absence. Lest he throws me out of the program for the sin of concealing a pregnancy, I needed, I reasoned, to prove myself indispensable. This led to many sleepless nights until I hit on a bright idea. ‘Why don't you,’ I said to Phil, ‘figure out how these new ‘major tranquillizers,’ that we rely so much on, do their work? That could serve as your PhD thesis, should be fairly easy to do (I was very naïve), and would guarantee them wanting me back.’

Phil liked the plan. He conceived the idea that the new drugs – there were six or seven available at that time (several phenothiazines plus haloperidol) – had to enter neurons through the cell membrane, and, since the membranes of red cells were similar to those of neurons, why not visualize under the electron microscope what these drugs did to red cell membranes.

Our baby was born and I was on leave from work for a few months so, every morning, I rolled up Phil's shirt sleeve, took his blood in a specially marked vial, which he walked down to the lab, made a blood smear on a slide, mixed different doses of the various antipsychotic drugs, and other drugs as well, into the blood, and looked to see what the microscope showed. He took photographs of the red cell membranes under the influence of different drugs and we later hired one of my patients, who loved the precision that the work required and was excellent at it, to carefully measure the widths of Phil's red cell membranes.

The upshot of all this was that I wasn't thrown out of the program, that the patient/research assistant got well and became a successful New York artist, and Phil got his PhD after a few years. But he didn't discover how antipsychotic drugs worked. Instead, his PhD work led to the membrane theory of anesthetic action (Seeman, 1966).

Before Phil graduated, Carlsson and Lindqvist (1963) had reported that chlorpromazine and haloperidol increased the level of catecholamine metabolites by blocking monoaminergic receptors in the brain. They didn't specify which monoamine because it was not possible, at the time, to separate alpha or beta-adrenoreceptors from dopamine receptors. There were reasons, however, to implicate the dopamine pathway because Parkinson's disease had, in 1960, been linked to dopamine deficiency (Ehringer & Hornykiewicz, 1960), and the clinical side effects of the drugs Phil was trying to study – tremor, rigidity, and akinesia, – mimicked Parkinson's. Furthermore, dopamine-mimetic drugs such as amphetamine were already known to induce psychotic symptoms (Connell, 1957).

As a result, dopamine was much discussed when Phil and I went to England so he could do a post-doc with his former mentor, Arnold Burgen, now at Downing College, Cambridge. Needless to say, there were many inspiring ‘Great Names’ in Cambridge, and Burgen figured among them. He had become a pioneer in the use of nuclear magnetic resonance in pharmacology (Aellig, 1990). Among other important lessons, Phil learned how to use radiolabelled irreversible antagonists to visualize membrane receptors and it was in Cambridge that he learned about the fast interconversion of different receptor states. At the end of the post-doc, he was appointed to the department of pharmacology at the University of Toronto.

This was 1967. The year before, Jacques van Rossum, a professor of pharmacology at the medical faculty of the University of Nijmegen in the Netherlands, had suggested something important:

‘When the hypothesis of dopamine blockade by neuroleptic agents can be further substantiated, it may have far going consequences for the pathophysiology of schizophrenia. Overstimulation of dopamine receptors could then be part of the aetiology. Obviously such an overstimulation might be caused by overproduction of dopamine, production of substances with dopamine actions (methoxy derivatives), abnormal susceptibility of the receptors, etc.’ (Van Rossum, 1966).

Receptors for neurotransmitters were being actively discussed but none, at that point, had yet been isolated. The first one, the nicotinic acetylcholine receptor, was identified in 1970 (Changeux, Kasai, & Lee, 1970) as a result of new biochemical methods radically changing the potential for receptor identification. One such powerful method was affinity labeling (Wold, 1977), the use of compounds that are structural homologs of the neurotransmitter and also possess a highly reactive molecular site that binds to the protein receptor.

Setting up his lab at the University of Toronto in the basement of the Fitzgerald Building, and following Changeux's lead, Phil set out to isolate the post synaptic membrane receptor for dopamine, the one that van Rossum suggested was blocked by the drugs, which were by then called neuroleptics. The following year, Zingales (1971) reported that the concentration of haloperidol in the plasma of successfully treated patients with psychosis was approximately 3 nanograms per milliliter of plasma (3 nmol). Because over 90% of haloperidol in plasma is bound to plasma proteins, the actual free concentration that enters the brain, Phil estimated, would be approximately 1 nmol. This was a problem for the radioactive tagging required to locate specific targets of haloperidol action. Because the drug needed to be diluted down to 1 nmol, the radioactive label needed to be extremely powerful. No such label existed at the time.

Phil had made friends with the brilliant Belgian pharmacologist who had synthesized haloperidol, Paul Janssen, who was later dubbed ‘the most important Belgian scientist’ (Anonymous, 2008). Phil leaned on Janssen to persuade the Institut National des Radioéléments (I.R.E.) Belgique to prepare radioactive haloperidol at the specificity required, which was 10.5 Curies per millimole.

This took years, during which time Phil and his team tried many approaches to pinpointing the mode of action of neuroleptics (the many failed attempts are outlined in Madras, 2013). Finally, the radioactive haloperidol from I.R.E. Belgique arrived. Phil mixed the labeled haloperidol with striatal brain tissue and measured whether radioactive molecules remained after washing. They did.

Besides a site being radioactively tagged, an important criterion of specificity was stereoselectivity – the configuration of the relevant molecule must fit the configuration of the target (McConathy & Owens, 2003). Phil obtained mirror image antipsychotic molecules (+butaclamol and −butaclamol), newly synthesized by Leslie Humber working at Ayerst, Canada. The first molecule was active, the second inactive (Bruderlein, Humber, & Voith, 1975). A specific antipsychotic target was confirmed when the identified site was blocked by +butaclamol to a significantly greater degree than it was by −butaclamol. The last step was to see which endogenous neurotransmitter had the most affinity for the identified site. When tested against noradrenaline, acetylcholine, serotonin, and dopamine, dopamine proved the winner. This meant that the antipsychotic receptor was a dopamine receptor (Seeman, Chau-Wong, Tedesco, & Wong, 1975).

The characterization of the ‘antipsychotic receptor’ as a dopamine receptor was an important step forward in understanding how drugs were able to control psychotic symptoms. Phil also showed that the clinically effective doses of all antipsychotic medications available at the time, regardless of their molecular structure, directly correlated with the drug's ability to displace radioactive haloperidol (Seeman, Lee, Chau-Wong, & Wong, 1976). The graph that accompanied this 1976 paper has been called, as recently as this year, ‘the most famous graph in schizophrenia therapeutics’ (Tricklebank et al., 2021).

Eventually, five different dopamine receptor types were identified in the brain. They are all G-protein coupled membrane receptors that, based on their signal transduction characteristics, are traditionally divided into two subfamilies (Seeman, 1980). Of the five receptors, D1, D4, and D5 were cloned in the Seeman laboratory (Sunahara et al., 1990, 1991; Van Tol et al., 1991).

Phil's receptor, the dopamine D2 receptor, was cloned in 1988 in the Civelli lab (Bunzow et al., 1988; Grandy et al., 1989a, 1989b). Grandy et al. (1989b) used *in situ* hybridization to map the gene to the 11q22–q23 junction (Grandy et al., 1989a).

Phil remained convinced all his life that the D2 receptor would one day prove critical to understanding the pathophysiology of schizophrenia. The receptor exists in the brain in two states – a state of high-affinity for dopamine [D2(High)] and a state of low-affinity (D2Low). The D2(High) state in animal models of schizophrenia leads to dopamine supersensitivity whether produced by brain lesions, drugs such as amphetamine or cocaine, prolonged social isolation, or gene deletions in key brain pathways (Seeman, 2011). In humans, dopamine supersensitivity is known to frequently emerge after the long term use of antipsychotics (Chouinard et al., 2017). But there is no reason to think that it could not be triggered, as in experimental animals, by genetic deviations or adverse exposures and circumstances at critical time periods of development. Phil believed that it was D2 supersensitivity that was responsible for psychotic symptoms in humans (Seeman, 2011; Seeman & Seeman, 2014).

Toward the end of his life, in collaboration with John Neumeyer, Phil tried to find a way of imaging the D2High state in humans, hoping to show that its presence correlated with psychotic symptoms (Inkster, Sromek, Akurathi, Neumeyer, & Packard, 2021; Subburaju, Sromek, Seeman, & Neumeyer, 2018, 2021).

He would have been thrilled to know that therapies for dopamine supersensitivity and the D2High state are gradually becoming possible (Kruyer et al., 2021).

## Conclusion

Small steps can lead to transformative change.
